# Human liver stem cells attenuate concanavalin A-induced acute liver injury by modulating myeloid-derived suppressor cells and CD4^+^ T cells in mice

**DOI:** 10.1186/s13287-018-1128-2

**Published:** 2019-01-11

**Authors:** Yanzhen Bi, Jiannan Li, Yonghong Yang, Quanyi Wang, Quanquan Wang, Xiaobei Zhang, Guanjun Dong, Yibo Wang, Zhongping Duan, Zhenfeng Shu, Tongjun Liu, Yu Chen, Kai Zhang, Feng Hong

**Affiliations:** 1 Beijing Artificial Liver Treatment & Training Center, Beijing Youan Hospital, Captial Medical University, Beijing, 100069 People’s Republic of China; 2 Department of General Surgery, The Second Hospital of Jilin University, Changchun, 130041 People’s Republic of China; 3 Institute of Liver Diseases, Affiliated Hospital of Jining Medical University, Jining, 272067 People’s Republic of China; 4 Department of Neuromuscular Disease, The Third Hospital of Hebei Medical University, Shijiazhuang, People’s Republic of China; 5 Institute of Immunology and Molecular Medicine, Jining Medical University, Jining, People’s Republic of China; 6 Shanghai Meifeng Biotechnology Co., Ltd, Shanghai, People’s Republic of China

**Keywords:** Acute liver injury, Human liver stem cells, MDSCs, CD4^+^ T cells, Concanavalin A

## Abstract

**Background:**

Acute liver failure (ALF) is a serious threat to the life of people all over the world. Finding an effective way to manage ALF is important. Human liver stem cells (HLSCs) are early undifferentiated cells that have been implicated in the regeneration and functional reconstruction of the liver. In this study, we aimed to evaluate the protective effects of the HLSC line HYX1 against concanavalin A (ConA)-induced acute liver injury.

**Methods:**

HYX1 cells were characterized by microscopy, functional assays, gene expression, and western blot analyses. We showed that HYX1 cells can differentiate into hepatocytes. We intraperitoneally injected HYX1 cells in mice and administered ConA via caudal vein injection 3, 6, 12, 24, and 48 h later. The effects of HYX1 cell transplantation were evaluated through blood tests, histology, and flow cytometry.

**Results:**

HYX1 cells reduced the levels of alanine transaminase (ALT), aspartate aminotransferase (AST), and total bilirubin (TBIL) in serum and dramatically decreased the severity of liver injuries. Mechanistically, HYX1 cells promoted myeloid-derived suppressor cell (MDSC) migration into the spleen and liver, while reducing CD4^+^ T cell levels in both tissues. In addition, HYX1 cells suppressed the secretion of proinflammatory cytokines, such as tumour necrosis factor-α (TNF-α) and interferon-γ (IFN-γ), but led to increased interleukin-10 (IL-10) production.

**Conclusions:**

These results confirm the efficacy of HLSCs in the prevention of the ConA-induced acute liver injury through modulation of MDSCs and CD4^+^ T cell migration and cytokine secretion.

**Electronic supplementary material:**

The online version of this article (10.1186/s13287-018-1128-2) contains supplementary material, which is available to authorized users.

## Background

The liver is the largest internal organ and performs very important functions, such as production of bile, storage of glycogen, and filter of harmful substances, among others. Liver diseases, especially acute liver failure (ALF), seriously threaten the life of people all over the world [1]. The factors that induce liver damage mainly include over-ingestion of some drugs, excessive drinking, viral infections, and autoimmune responses [2, 3]. ALF is often caused by autoimmune hepatitis and hepatitis B or C infection [4]. Concanavalin A (ConA) administration can significantly increase the liver transaminase levels and leads to acute hepatitis [5, 6]. The ConA-induced acute liver injury model is characterized by liver necrosis, activation of T lymphocytes, and overexpression of free radicals and pro-inflammatory cytokines [7, 8]. Therefore, this model is widely accepted for mechanistic studies of ALF.

ALF is characterized by acute onset, rapid progression, and a low survival rate. In addition, it often affects the function of multiple organ systems [4]. Consequently, finding an efficient way to treat/improve ALF is necessary and important. Nowadays, orthotopic liver transplantation and artificial liver systems are the usual treatment methods for ALF. Unfortunately, the possibility of liver transplantation is limited by the shortage of liver donors, immunological suppression, and high costs [9]. Stem cells, which can be derived from other tissues and expanded in vitro, have improved prospects for the treatment of many diseases [10, 11]. Liver stem cells (LSCs) are early undifferentiated cells which can proliferate efficiently, differentiate in multiple directions, and self-renew. Furthermore, they can also be involved in the functional regeneration of the liver [12, 13]. With the development of isolation and culture techniques for stem cells, LSCs might provide a new way to treat ALF [14, 15].

Myeloid-derived suppressor cells (MDSCs) are heterogeneous immature myeloid cells and characterized by the ability to suppress the proliferation and cytotoxicity of T cells and induce the expansion of regulatory T cells (Treg), which play an important role in hepatic protection [16]. The liver has been shown to be a site for MDSC accumulation that is associated with hepatic diseases [17]. Patients infected by hepatic viruses present increased levels of MDSCs in the peripheral blood [18]. Previous studies have also reported that CD4^+^ T lymphocytes increased ConA-induced acute liver injury [19, 20]. However, MDSCs can suppress and modulate the phenotype of CD4^+^ T cells [17].

In the present study, we investigated the protective effect of human liver stem cells (HLSCs) against ConA-induced acute liver injury and further analysed their mechanisms of action in this murine model of ALF.

## Methods

### Materials

Human liver stem cells 1 (HYX1) were provided by Professor Hong Feng. ConA was purchased from Sigma-Aldrich (Shanghai, P. R. China). Dulbecco’s modified Eagle medium (DMEM) and foetal bovine serum (FBS) were purchased from Gibco (Gran Island, NY, USA). Indocyanine green (ICG) and periodic acid-schiff (PAS) were purchased from Sigma-Aldrich (Shanghai, P. R. China). Periodic acid and sulfuric acid were purchased from Beijing Chemical Works (Beijing, P. R., China). Paraformaldehyde, dimethyl sulfoxide (DMSO), and hepatocyte growth factor (HGF) were obtained from Beijing Huafeng United Technology Co., Ltd. (Beijing, P. R., China).

### Isolation and culture of HYX1 cells

Human liver specimens (20 to 50 g) obtained from Jining Medical University Affiliated Hospital were first perfused in situ for 10 min with 200 to 300 mL of EGTA buffer and collagenase type IV (0.05%) in DMEM until the tissue became soft and showed signs of dissolution. The tissue was minced, and the cell suspension was centrifuged twice at 50 × *g* for 2 min at 4 °C. The supernatant was collected and centrifuged at 150 × *g* for 8 min at 4 °C. The resultant cell pellet was resuspended in DMEM and centrifuged at 150 × *g* for 5 min at 4 °C. Finally, the pelleted cells containing crude HLSCs were suspended in PBS for purification in density gradients made of 50%, 70%, and 90% Percoll (Sigma-Aldrich) and cell suspension. To spread layer by layer from the bottom of the tube, place the cell suspension on the top layer. The preparation was centrifuged at 350 × *g* for 20 min at 4 °C. The interface between the 50% and 70% Percoll was decanted to a tube and centrifuged at 350 × g for 5 min. The cell pellet was resuspended in DMEM and centrifuged twice at 1200 rpm for 5 min at 4 °C.

The purified HLSCs were collected and used for culture in six-well plates in DMEM supplemented with 10% FBS, 100 U/ml penicillin, 100 μg/ml streptomycin, 1 mg/l insulin, and 1 × 10^7^ mol/l dexamethasone. These were cultured for 2–3 weeks with the medium changed twice a week. When colonies became visible, they were encircled with cloning rings and subcultured to an individual well of a six-well plate. The expanded cells were taken for assessment of markers of hepatic stem cells. The human liver stem cells isolated are named HYX1, which can be currently subcultured to 50 generations. The initial batch of HYX1 cells was cultured for 20 days, and the cells were photographed after the 10th passage under a phase-contrast microscope (CKX31, Olympus, Tokyo, Japan). The high-resolution morphology of HYX1 cells was examined by transmission electron microscopy (TEM, JEOL, Tokyo, Japan). Thereafter, cells were transferred to T-75 flasks. At confluence, cells were taken for experiments.

### ICG uptake assays

ICG uptake assays were used to analyse the hepatic function of HYX1 cells. Briefly, HYX1 cells (10th passage) were treated with 1 mg/ml ICG at 37 °C for 1 h. The cells were washed twice with phosphate-buffered saline (PBS) and resuspended with DMEM, low glucose (1000 mg/L) containing 10% FBS. The cells were then observed under a CKX31 microscope.

### PAS staining

PAS staining was used to estimate the glycogen storage functions of the cells. HYX1 cells (10th passage) were treated with 4% paraformaldehyde for 10 min, washed with PBS, and air dried. The cells were then dealt with in 1% periodic acid. Finally, the cells were stained with PAS for 30 min at room temperature, washed with sulfuric acid in PBS, and air dried. The cells were analysed under a CKX31 microscope.

### Reverse transcription-polymerase chain reaction (RT-PCR) for HYX1 cells

RT-PCR was performed to analyse expression of albumin (*ALB*), alpha fetoprotein (*AFP*), and cytokeratin (*CK*)*7*, *CK8*, and *CK19* in HYX1 cells. Total RNA from HYX1 cells was isolated using a RNAiso kit (Takara, Otsu, Japan). The Moloney murine leukaemia virus reverse transcriptase (M-MLV) was used to synthetize cDNA. The resultant cDNA was then subjected to PCR amplification and separated by electrophoresis; the DNA signals on the gel were imaged. The sequences for the primers are listed in Table 1.

**Table 1:** RT-PCR primer sequences

Gene	Forward primer	Reverse primer
*AFP*	5′-GCAAAGCTGAAAATGCAGTTGA-3′	5′-GGAAAGTTCGGGTCCCAAAA-3′
*CK7*	5′-TGAATGATGAGATCAACTTCCTCAG-3′	5′-TGTCGGAGATCTGGGACTGC-3′
*CK8*	5′-TCATAGACAAGGTACGGTTCC-3′	5′-GCCTAAGGTTGTTGATGTAGC-3′
*CK19*	5′-TCGACAACGCCCGTCTG-3′	5′-CCACGCTCATGCGCAG-3′
*ALB*	5′-CTGAGCAAAGGCAATCAACA-3′	5′-CACAGTCTGCTGAGGTTGGA-3′

### Western blot analysis

Western blot analysis was performed to analyse the ability of HYX1 cells to differentiate in hepatic cells. HYX1 cells were treated with 2% DMSO and HGF (20 ng/mL) for 5 days in 2% DMSO+HGF group, and the control group was added to an equal volume of PBS for 5 days. Proteins were extracted from HYX1 cells. The protein concentrations were determined using a bicinchoninic acid (BCA) protein assay kit (Beyotime, Nanjing, P. R. China). Equal amounts of proteins were separated by sodium dodecyl sulfate-polyacrylamide gel electrophoresis (SDS-PAGE) and transferred to polyvinylidene difluoride (PVDF) membranes. The membranes were blocked in 5% skim milk and incubated with primary antibodies against ALB (1:1000 dilution, Abcam, Cambridge, UK) and CK19 (1:1000 dilution, Abcam) and HRP-conjugated secondary antibodies (1:3000; Boster, Wuhan, China). Finally, the immunoreactive signals were detected using an enhanced chemiluminescence (ECL) plus kit (Millipore, Temecula, CA, USA).

### Mice and treatment

Male C57BL/6 mice weighing 22–25 g (Jinan Pengyue Laboratory Animal Breeding Co., Ltd., Jinan, China) were maintained according to the National Institutes of Health guidelines for the care and use of animals, approved by the Institutional Animal Care and Use Committee of Jining Medical University Affiliated Hospital, China. Mice were kept in the Animal Care Facility of Jining Medical University with a 12 h light–dark cycle at constant temperature. Mice had free access to tap water during the study period.

**Fig. 1:**
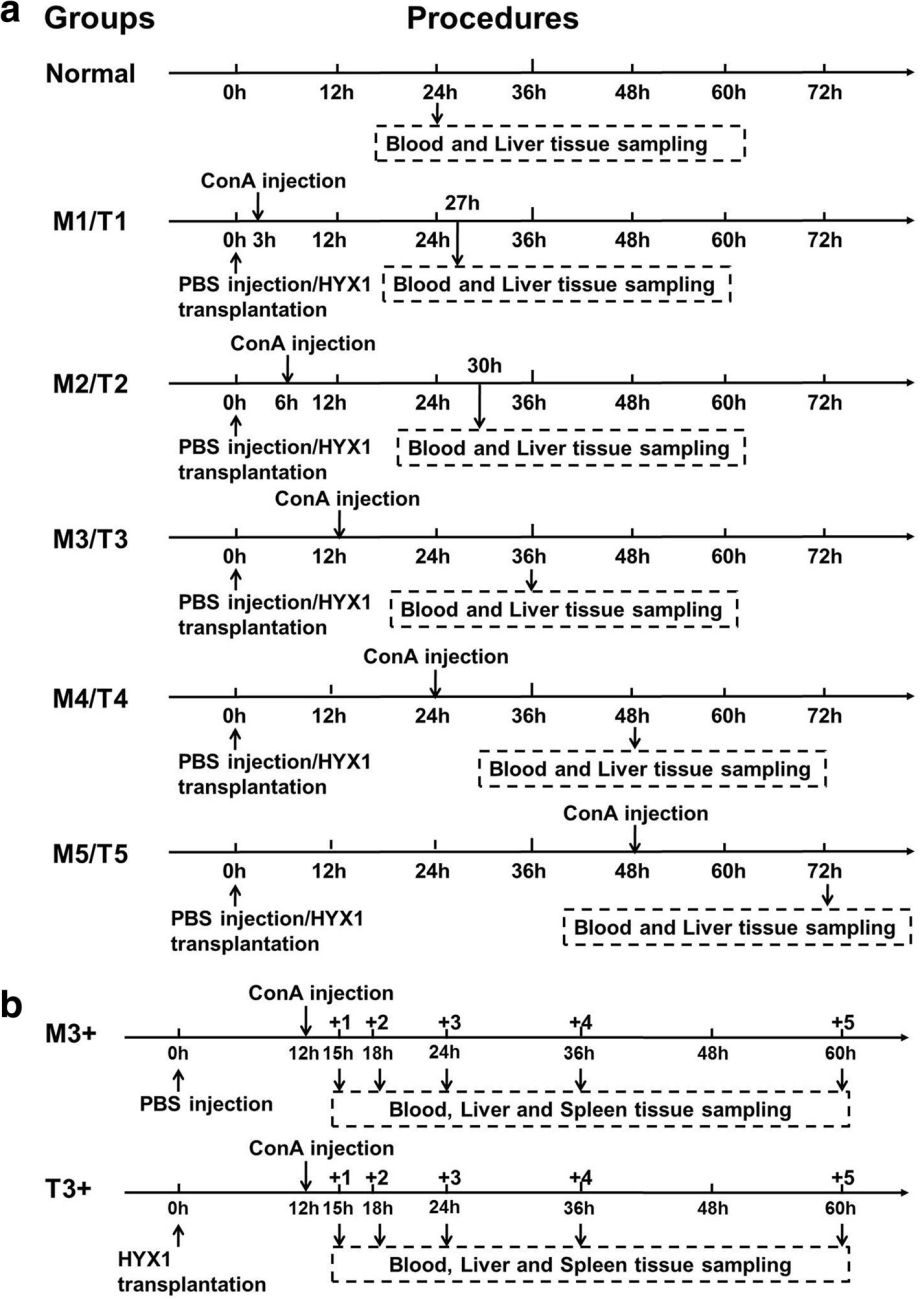
Schematic outline of the experimental groups. **a** Initial grouping of the mice; mice were sacrificed for sample collection 24 h after ConA treatment. **b** In the + 1, 2, 3, 4, and 5 groups, mice were sacrificed for sample collection 3, 6, 12, 24, and 48 h, respectively, after ConA injection

In this study, 66 mice were randomly divided into three groups: normal (*n* = 6), ConA (labelled as M, *n* = 30), and HYX1+ConA (labelled as T, *n* = 30). In the normal group, mice were injected with 200 μL PBS via tail vein. In the M group, mice were injected with 12 mg/kg ConA via tail vein after 200 μL PBS were administered via intraperitoneal (IP) injection. In the T group, mice were injected with 2.0 × 10^6^ HYX1 cells (10th passage) via IP injection before treatment with ConA. Both the M and T groups were further divided into subgroups, 1 through 5, injected with PBS (group M) or HYX1 cells (group T) 3, 6, 12, 24, 48 h before treatment with ConA (Fig. 1a). For the M and T groups, 24 h post-ConA injection, blood was collected and the mice were sacrificed for liver sample collection (Fig. 1a). For normal group, blood serum and liver specimens were collected 24 h post-PBS injection. Blood serum was evaluated for biochemical parameters, and liver tissues were analysed for histopathology. We found that HYX1 cell injection 6 or 12 h before treatment with ConA (T2 or T3 groups) had the best effect against acute liver injury [21]. Therefore, based on the results from the M3 and T3 subgroups, we established two more groups called M3+ and T3+ injected with PBS or HYX1 cells, respectively, 12 h before treatment with ConA; each group contained five different subgroups (*n* = 6) named M3 or T3 +1, + 2, + 3, + 4, and + 5, in which mice were sacrificed 3, 6, 12, 24, and 48 h, respectively, after ConA injection (Fig. 1b). At each time point post-ConA injection, blood was collected, and the mice were sacrificed for spleen and liver sample collection. Blood serum was evaluated for biochemical parameters and inflammatory factors. Liver and spleen samples were analysed for histopathology and potential changes of the MDSCs and CD4^+^ T cell populations.

### Blood serum analysis

Before the animals were sacrificed, blood was collected from the orbital plexus and then centrifuged at 3000 rpm for 10 min to obtain the serum. The levels of alanine transaminase (ALT), aspartate transaminase (AST), and total bilirubin (TBIL) were detected by using an automated analyser (AU5800, Beckman Coulter, Brea, CA, USA).

### Flow cytometry analysis

For flow cytometry, fluorescently labelled antibodies against MDSCs (defined as CD11b+ Gr-1+ cells) and CD4 markers were obtained from eBioscience (San Diego, CA, USA) and used at a 1:100 dilution. Cell suspensions from the liver and spleen were prepared after the animals were sacrificed. For phenotype staining, cells were washed twice with PBS containing 1% FBS and 0.1% NaN_3_. The cells were then incubated for 60 min at 4 °C with the following anti-mouse antibodies according to standard procedures: CD11b-fluorescein isothiocyanate (FITC), Gr-1-allophycocyanin (APC), CD3-FITC, and CD8a-PerCP Cyanine5.5. After two washes with PBS, the cells were analysed using a FACSCalibur (Becton Dickinson, San Jose, CA, USA). An isotype control was used for each antibody.

### Haematoxylin and eosin staining

The liver tissues were collected at the same location as the flow cytometry samples, rinsed with PBS, fixed with 4% formalin, and embedded in paraffin. The samples were then sectioned and stained with haematoxylin and eosin (H&E).

### Quantitative PCR (qPCR) analysis of mouse liver samples

Total RNA was extracted from 30 mg of mouse liver. The RNA (5 μg) was reverse transcribed into cDNA as described above and analysed in triplicate by qPCR using a SYBR green qPCR Master Mix (Annoron, Roche, Beijing, China) on the LightCycler® 480 Real-Time PCR System (Annoron, Roche, Beijing, China). The sequences of the qPCR primers are listed in Table 2.

**Table 2:** Quantitative PCR primer sequences

Gene	Sequence (5′-3′)	Product (bp)
*GAPDH*	F: CTAGAGAGCTGACAGTGGGTAT	49
	R: AGACGACCAATGCGTCCAAA	
*TNF-α*	F: CCCTCACACTCAGATCATCTTCT	61
	R: GCTACGACGTGGGCTACAG	
*IFN-γ*	F: TCGGTAACTGACTTGAATGTCCA	93
	R: TCGCTTCCCTGTTTTAGCTGC	
*IL-10*	F: TCAAGGCGCATGTGAACTCC	176
	R: GATGTCAAACTCACTCATGGCT	

### Enzyme-linked immunosorbent assay (ELISA)

Mouse serum samples were collected from the orbital plexus. The levels of TNF-α, IFN-γ, and IL-10 were detected by using ELISA kits according to the manufacturer’s instructions (R&D Systems, Minneapolis, MN, USA).

### Statistical analysis

All results were presented as the mean ± standard error of mean (SEM) and analysed using the SPSS 13.0 statistical software (IBM, Armonk, NY, USA). The differences between each group were compared by one-way analysis of variance (ANOVA) or Kruskal-Wallis test as appropriate. *P* < 0.05 (*) indicated statistical significance; *P* < 0.01 (**) and *P* < 0.001 (***) indicated high statistical significance.

## Results

### Morphologic characterization of HYX1 cells

**Fig. 2:**
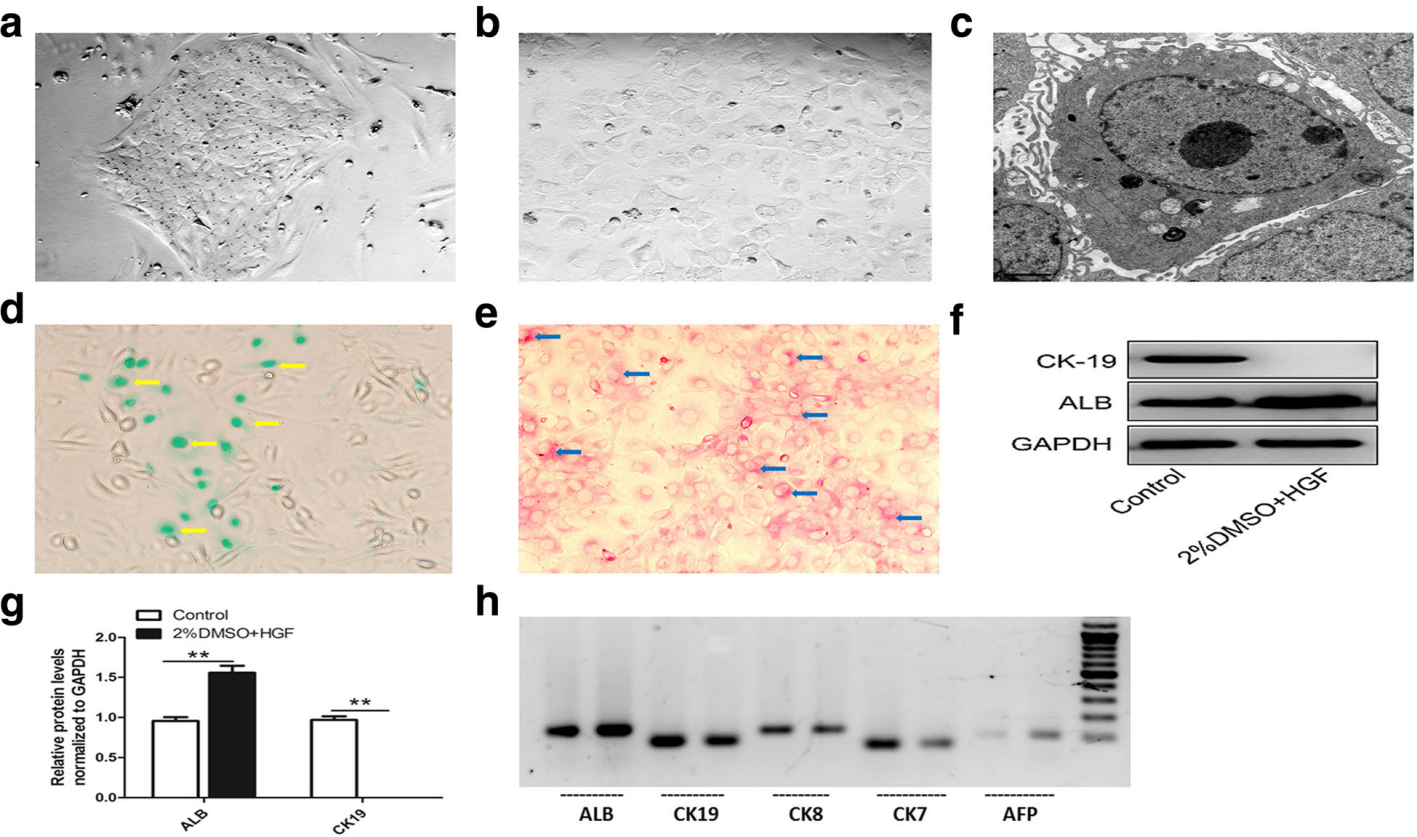
Characterization of HYX1 cells. **a** During the first 20 days in culture, the cells formed clusters and appeared round. **b** In subculture, the cells spread out and looked flattened and larger (original magnification × 100). **c** Representative TEM image of an HYX1 cell. **d** ICG uptake assay showed pale green ICG-positive cells (as indicated by the yellow arrows). **e** PAS staining of HYX1 showed positive cells with cytoplasm appeared red (as indicated by the blue arrows). **f**, **g** HYX1 cells were treated with DMSO + HGF or PBS (control group) for 5 days and western blot analysis of the indicated proteins. **h** RT-PCR for the indicated genes. Data are presented as mean ± SEM; compared with control group, *P < 0.05, **P < 0.01, as determined by *t* test

During the first 20 days of culture, HYX1 cells that were attached on culture plates appeared small and round, forming colonies (Fig. 2a). In subsequent subculture, the cells looked enlarged and more flattened, resembling hepatocytes (Fig. 2b). TEM examination showed that HYX1 cells were small, oval, and mostly occupied by oval nuclei, with large nucleus-to-cytoplasm ratios. The major organelles, such as the mitochondria and ribosomes, were underdeveloped, resembling the characteristics of undifferentiated cells (Fig. 2c). ICG uptake assays showed pale green ICG-positive cells (Fig. 2d) and PAS staining showed that a large number of positive cells with cytoplasm appeared red (Fig. 2e). This indicated that HYX1 cells possessed the functions of normal liver cells.

Next, we evaluated the expression of hepatocyte and biliary epithelial cell markers by HYX1 cells. RT-PCR showed that these cells expressed the hepatocyte marker genes *ALB*, *CK8*, and *AFP* (though the expression of the latter was weak), as well as the cholangiocyte marker genes *CK7* and *CK19* (Fig. 2h). Western blots demonstrate expression of albumin and CK19 by HYX1 cells. Upon exposure to DMSO and HGF, the expression of albumin was elevated, while that of CK19 was lost (Fig. 2f, g). This indicates that HYX1 can be induced into hepatocytes.

### Biochemical analysis after HYX1 cell transplantation

**Fig. 3:**
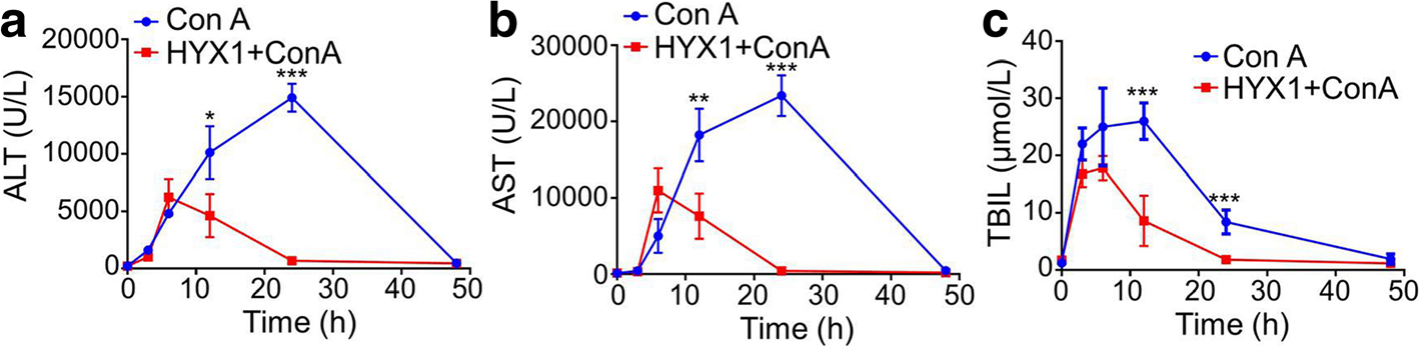
Biochemical evaluation of **a** ALT, **b** AST, and **c** TBIL in serum from the M3+ (blue) and T3+ (red) mice. Data are presented as mean ± SEM (*n* = 6; *P < 0.05, **P < 0.01, ***P < 0.001), as determined by ANOVA tests or *t* test; ns denotes *P* > 0.05

To evaluate the therapeutic potential of HYX1 cell transplantation against ConA-induced acute liver injury, the serum levels of ALT, AST, and TBIL were analysed in the ConA and HYX1 + ConA groups. In a previous paper, we have already reported that the best protection against acute liver injury in mice is achieved when ConA was injected 6 or 12 h after intraperitoneal transplantation of HYX1 cells [21] (Additional file 1: Figure S1). In Fig. 3, further research indicated that significantly lower levels of ALT, AST, and TBIL were observed 12 and 24 h after HYX1 transplantation in the T3+ group compared with the M3+ group. The levels of ALT, AST, and TBIL in the T3+ group were the highest at 6 h, and quickly dropped to normal levels by 24 h. In contrast, the M3+ group levels peaked at 12 (TBIL) or 24 (ALT and AST) h, and gradually decreased to normal by 48 h. These results suggest that HYX1 intraperitoneal transplantation has a protective effect on acute liver injury.

### Histopathology after HYX1 cell transplantation

**Fig. 4:**
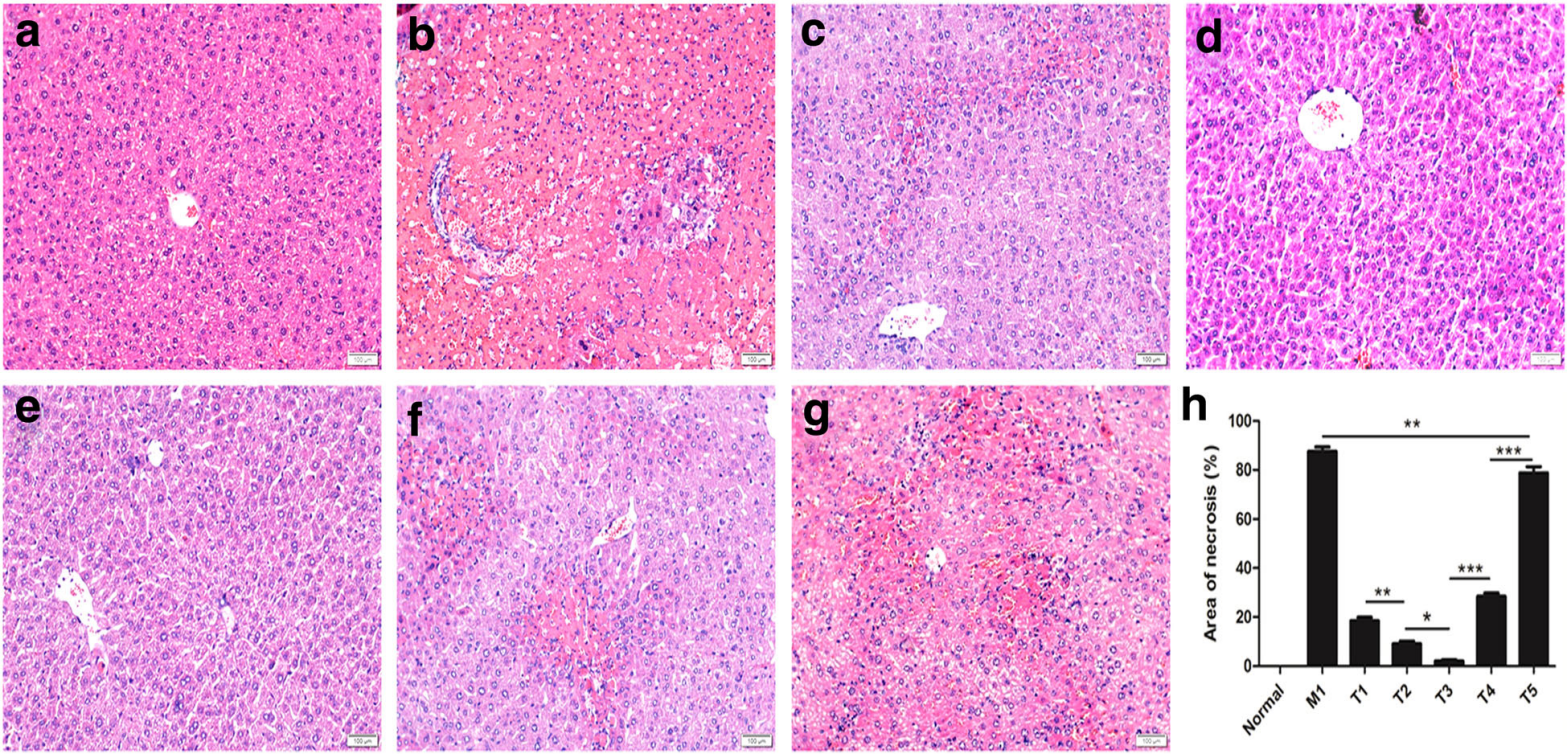
Representative images of haematoxylin and eosin staining of the liver and quantitation of necrosis area in the normal, M, and T groups. **a** Normal group; **b** M1 group; **c**–**g** T1, T2, T3, T4, and T5 groups; **h** necrosis area of each group. Data are presented as mean ± SEM (*n* = 6; *P < 0.05, **P < 0.01, ***P < 0.001), as determined by ANOVA tests; ns denotes *P* > 0.05. Scale bar = 100 μm

H&E staining showed no signs of liver cell degeneration, necrosis, or inflammatory infiltration in the normal group (Fig. 4). In the M groups, hepatocellular congestion and oedema were obvious and the liver parenchyma and bile ducts were infiltrated with inflammatory cells. In addition, the hepatic lobule was damaged and large areas of necrosis were observed (Fig. 4). On the other hand, liver injury was not obvious in the T1, T2, or T3 groups and only mild inflammatory cell infiltration was observed in the T2 and T3 groups. In the T1 and T4 groups, hepatic cell congestion, oedema, inflammatory infiltration, punctate, and flaky necrosis were markedly relieved compared to the M4 group. However, the T5 group showed hepatocellular congestion and oedema, considerable inflammation, and a small amount of punctate and flaky necrosis, while the basic hepatic lobules remained intact. These changes were consistent with the differences observed in the biochemical data (Additional file 1: Figure S1).

**Fig. 5:**
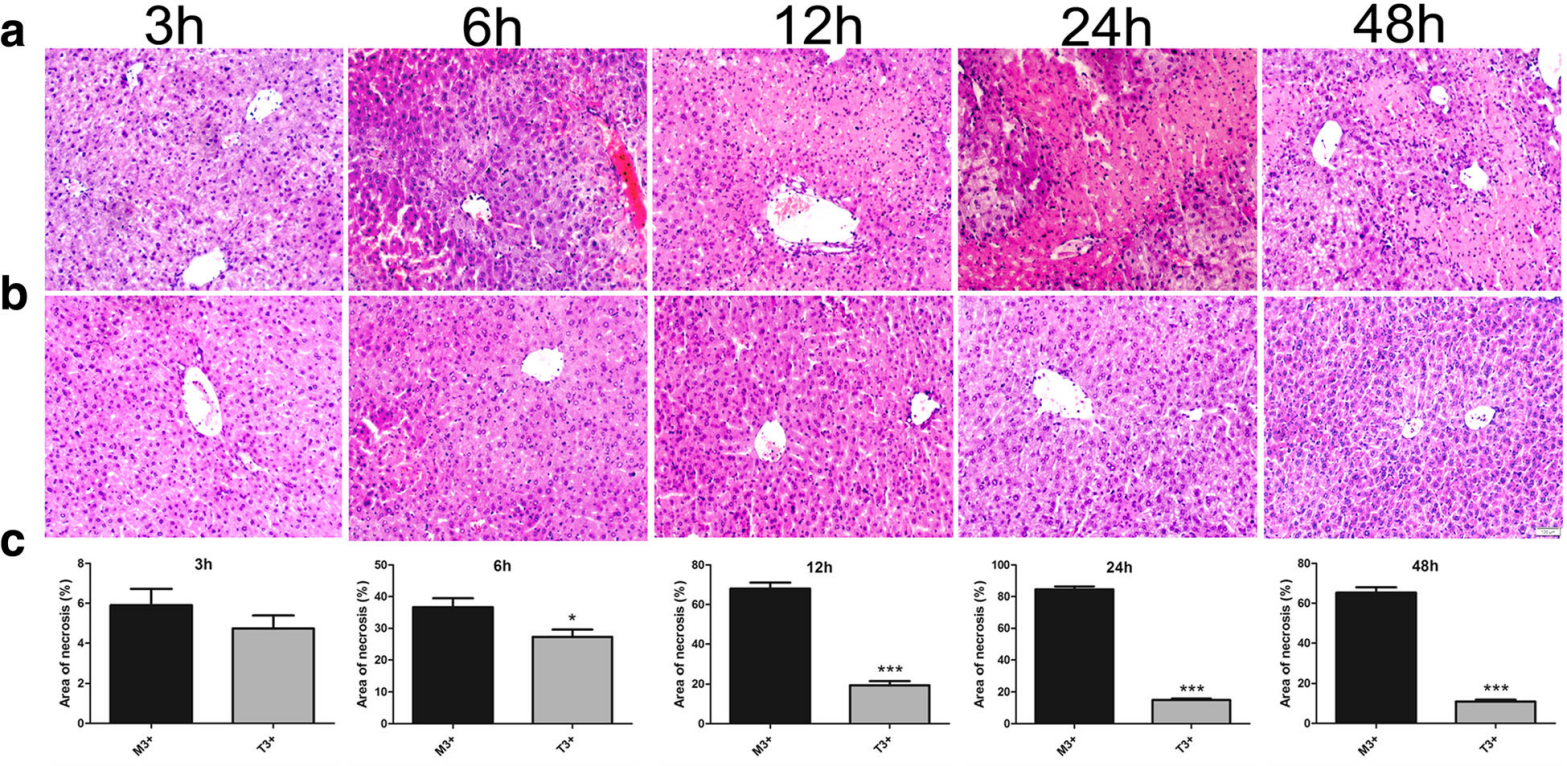
Representative images of haematoxylin and eosin staining of the liver and quantitation of necrosis area in M3+ and T3+ groups. **a** M3+ group; **b** T3+ group; **c** necrosis area of each group. Data are presented as mean ± SEM (*n* = 6; *P < 0.05, **P < 0.01, ***P < 0.001), as determined by *t* test; ns denotes *P* > 0.05. Scale bar = 100 μm

Furthermore, we found that hepatocyte necrosis worsened from 3 to 48 h in the M3+ group (Fig. 5). Although the levels of ALT, AST, and TBIL had dropped to normal at 48 h (Fig. 3), H&E staining showed that the necrosis was still evident in the M3+ group at this time point (Fig. 5). As shown in Fig. 5, there were only mild hepatocellular congestion, oedema, and inflammatory infiltration in the T3+ group. These results indicate that HYX1 intraperitoneal transplantation is protective against ConA-induced acute liver injury.

### Modulation of MDSCs and CD4^+^ T cells after HYX1 cell transplantation

**Fig. 6:**
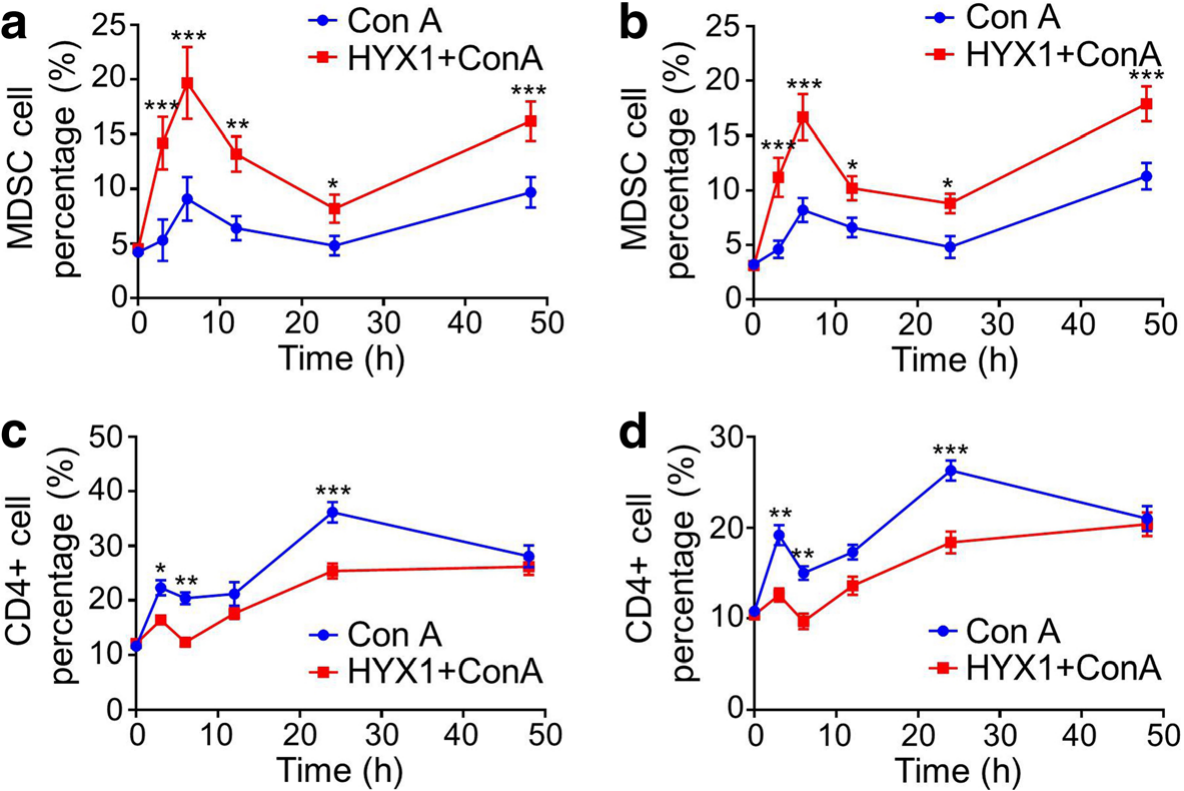
Changes in MDSCs (**a**, **b**) and CD4^+^ T cell levels (**c**, **d**) in the liver (**a**, **c**) and spleen (**b**, **d**) in M3+ and T3+ groups of mice. Data are presented as mean ± SEM (*n* = 6); *P < 0.05, **P < 0.01, ***P < 0.001, as determined by ANOVA tests or *t* test; ns denotes *P* > 0.05

To evaluate the mechanisms of HYX1 cells against ConA-induced acute liver injury, we performed flow cytometry analyses of MDSCs and CD4^+^ T cells in liver and spleen samples from the different groups (Fig. 6). The percentage of MDSCs and CD4^+^ T cells increased immediately after ConA injection in both the liver and spleen. The percentage of CD4^+^ T cells increased at 3 h and then decreased with the increased percentage of MDSCs in both the liver and spleen (M3+1, T3+1). At 6 h, the percentage of MDSCs reached its peak, while the percentage of CD4^+^ T cells dropped to its lowest level in both tissues (M3+2, T3+2). Then, the percentage of MDSCs decreased gradually and dropped to its lowest levels at 24 h, while the percentage of CD4^+^ T cells increased gradually and reached its peak at the same time in both the liver and spleen (M3+3, M3+4 and T3+3, T3+4). After 24 h after ConA injection, the percentage of MDSCs gradually increased again, accompanied by a continuous decline in the percentage of CD4^+^ T cells in both the liver and spleen (M3+4, M3+5 and T3+4, T3+5). Interestingly, the fluctuations in the percentage of CD4^+^ T cells were consistent with the levels of serum ALT, AST, and TBIL, which were contrary to the dynamics of MDSC presence from 3 to 48 h post-ConA injection (Figs. 3 and 6).

During the treatment process of HYX1 cells against ConA-induced acute liver injury, the percentages of MDSCs were significantly higher in the HYX1 + ConA group mice (T3+) than in the ConA group mice (M3+) from 3 to 48 h (*P* < 0.05), while, conversely, the percentages of CD4^+^ T cells were significantly lower in HYX1 + ConA group mice (T3+1, T3+2, T3+4) than in ConA group mice (M3+1, M3+2, M3+4) for both the liver and spleen from 3 to 24 h (*P* < 0.05; Fig. 6).

### Detection of TNF-α, IFN-γ, and IL-10 serum protein levels by ELISA after HYX1 cell transplantation

**Fig. 7:**
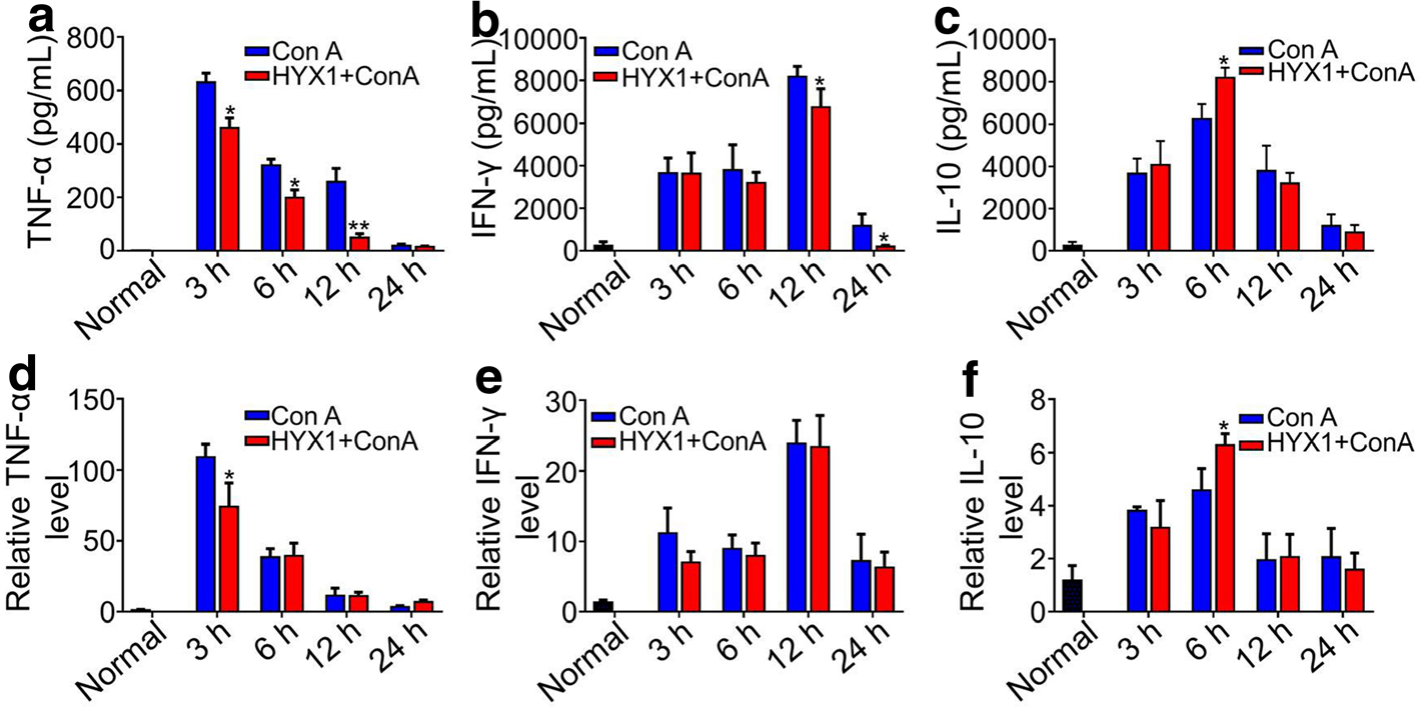
The protein levels of TNF-α, IFN-γ, and IL-10 in serum were quantified by ELISA (**a**–**c**) in the M3+ and T3+ groups of mice. The mRNA levels of *TNF-α*, *IFN-γ*, and *IL-10* in the liver were quantified by qPCR (**d**–**f**) in groups of M3+ and T3+ mice. Data are presented as mean ± SEM (*n* = 6); *P < 0.05, **P < 0.01, as determined by ANOVA tests; ns denotes *P* > 0.05

Next, we investigated the levels of pro- and anti-inflammatory cytokines in the serum of the different groups of mice. The levels of serum TNF-α (T3+1, T3+2, and T3+3) and IFN-γ (T3+3 and T3+4) of mice in the T3+ groups were significantly lower than those in the M3+ groups (*P* < 0.05; Fig. 7a, b), while the levels of IL-10 in the T3+2 group mice were significantly higher at 6 h compared to those of the M3+2 group mice (*P* < 0.05; Fig. 7c).

### Detection of TNF-α, IFN-γ, and IL-10 mRNA levels in liver by qPCR after HYX1 cell transplantation

We further investigated the effect of HYX1 cells on the expression of *TNF-α*, *IFN-γ*, and *IL-10* in liver. The results showed that levels of *TNF-α* decreased significantly in the T3+1 group compared to the M3+1 group (*P* < 0.05; Fig. 7d). Compared with the M3+ groups (M3+1, M3+2, M3+4), the levels of *IFN-γ* in the liver were also reduced in the T3+ groups (T3+1, T3+2, T3+4), but the differences were not statistically significant (*P* > 0.05; Fig. 7e). Furthermore, the levels of *IL-10* in the T3+2 group were significantly higher than those of the M3+2 group (*P* < 0.05; Fig. 7f).

## Discussion

ALF, often caused by chronic hepatitis and autoimmune responses, seriously endangers people’s lives. Even though medical advances have improved the health of patients with ALF, finding efficient treatment methods for this disorder is still a substantial challenge. In the present study, we used ConA to induce acute liver injury in mice. ConA is a lectin purified from *Canavalia ensiformis* [22]. The ConA-induced acute liver injury mouse model is the most commonly used animal model for the study of ALF. ConA can specifically activate CD4^+^ T cells and natural killer T lymphocytes, which can lead to acute liver injury [23]. Different laboratories use different dosages of ConA to induce the acute liver injury [8, 24, 25]. Therefore, we first investigated the appropriate ConA dose for this animal model in our facilities. We found that when 12 mg/kg ConA were used, the liver injury was serious and obvious and there was no animal death at 24 h post-ConA injection.

Nowadays, LSCs have gained increased attention for the treatment of liver injury due to their specific functional properties, which are similar to those of normal liver cells, and their ability to be expanded and cultured in large quantities in vitro. In addition, LSC treatment has many other advantages, such as low cost and mild immune rejection responses. Our group have successfully isolated human liver stem cells from liver tissues and developed a cell line (HYX1) which can be stably passed up to 50 generations [21].

In this study, we show that HYX1 cells cultured have the properties of hepatocytes. TEM examination demonstrated the undifferentiated characteristics of HYX1 cells, including large nucleus-to-cytoplasm ratio and underdeveloped organelles. ICG is a water soluble anionic complex that is uptaken by liver cells only and is used to evaluate hepatocyte function [26]. Hepatocytes are very important for glycogen storage and synthesis in vivo. Positive PAS staining is another marker of hepatocyte function. We found that HYX1 cells were stained by ICG and PAS, indicating that they had at least some of the functions of hepatocytes. Furthermore, the RT-PCR results showed that HYX1 express markers of both hepatocytes and bile duct epithelial cells. When HYX1 cells were treated with 2% DMSO and HGF, the expression of ALB was increased and the expression of CK19 was markedly reduced. This indicated that HYX1 cells could be induced by DMSO and HGF to differentiate into hepatocytes.

We have previously conducted an experiment to study the changes caused by HYX1 transplantation alone. We found that there was almost no changes in serum ALT, AST and TBIL in mice 24 h, 48 h, and 72 h after HYX1 transplantation alone, compared with normal mice (Additional file 1: Figure S4). The results of present study showed that HYX1 cell transplantation provides a protective effect against ConA-induced acute liver injury. Biochemical analyses indicated that HYX1 treatment reduced the serum levels of ALT, AST, and TBIL, especially when HYX1 cells were transplanted 6 or 12 h before treatment with ConA. However, when HYX1 cells were transplanted 48 h before ConA treatment, we did not observe a liver-protective effect. Histological examination also revealed that the liver injuries were mild in the T2, T3, and T4 groups (especially in T2 and T3). The results of our study are similar to those of Herrera et al, in which the HLSCs prevented liver injury by modulating the expression of cytokines with liver regenerative properties [27].

As we all know, implantation is critical for stem cell therapy [27], so we made a tracer experiment after HLSC transplantation into normal and liver-damaged mice. The results showed that the labeled HLSC transplanted from the tail vein could be colonized into the liver of mice with liver injury, but could not be colonized into the liver of normal mice. However, the labeled HLSC transplanted by intraperitoneal were not found in host liver with or without injury (Additional file 1: Figure S2). In addition, to identify whether there are any long-term protective effect after HSLC/ConA due-administration, We gave the mice a second injury 50 hours later. Interestingly, the recipients were still protected by donor cells (Additional file 1: Figure S3). These evidences indicate that HYX1 cells protect against liver injury by paracrine rather than implantation.

To further study the mechanisms of HYX1 cells against ConA-induced acute liver injury, we evaluated the levels of MDSCs and CD4^+^ T cells in the liver and spleen of the different groups. It has been reported that MDSCs could limit tissue injury during acute hepatitis and regulate immune responses by preventing T cell function [17, 28]. On the other hand, CD4^+^ T cells have been confirmed to increase the ConA-induced acute liver injury [19, 20]. In our study, HYX1 cells significantly increased the presence of MDSCs and decreased the levels of CD4^+^ T cells in the spleen and infiltrated in the liver. These results demonstrate that HYX1 cells can inhibit ConA-induced acute liver injury through the regulation of MDSCs and CD4^+^ T cells.

The expression of inflammatory cytokines in liver tissues, such as TNF-α, IFN-γ, and IL-10, was also detected. Our results indicated that the protective role of HYX1 cell treatment in ConA-induced ALF is also associated with the reduction of *TNF-α* and *IFN-γ* and increase of *IL-10* levels.

## Conclusions

In conclusion, this study demonstrated the efficacy of HLSCs in the prevention of the ConA-induced acute liver injury through the modulation of MDSCs and CD4^+^ T cell migration and cytokine secretion. Our study provides support for the use of HLSCs for the treatment of ALF.

## Additional file

Additional file 1: Biochemical evaluation in the serum and the detection of tracing of transplanted cells. Supplementary data: supplementary materials and methods and supplementary figures. **Supplementary Figure 1:** Biochemical evaluation of (a) ALT, (b) AST, and (c) TBIL in the serum from groups of normal, M (ConA) and T (HYX1 + ConA) mice. **Supplementary Figure 2:** Tracing of transplanted cells. **Supplementary Figure 3:** Long-term protective effect of HYX1 on a 2nd ConA-induced liver injury. **Supplementary Figure 4:** Biochemical evaluation of ALT, AST and TBIL in the serum of mice with HYX1 transplantation alone. (ZIP 27721 kb)

## Abbreviations

AFP: Alpha fetoprotein
ALB: Albumin
ALF: Acute liver failure
ALT: Alanine transaminase
AST: Aspartate aminotransferase
BCA: Bicinchoninic acid
CK: Cytokeratin
ConA: Concanavalin A
DMEM: Dulbecco’s modified Eagle medium
DMSO: Dimethyl sulfoxide
ECL: Enhanced chemiluminescence
ELISA: Enzyme-linked immunosorbent assay
FBS: Foetal bovine serum
H&E: Haematoxylin and eosin
HGF: Hepatocyte growth factor
HLSCs: Human liver stem cells
HYX1: Human liver stem cells 1
ICG: Indocyanine green
IFN-γ: Interferon-γ
IL-10: Interleukin-10
IP: Intraperitoneal
LSCs: Liver stem cells
MDSCs: Myeloid-derived suppressor cells
M-MLV: Moloney murine leukaemia virus reverse transcriptase
PAS: Periodic acid-Schiff
PBS: Phosphate-buffered saline
PVDF: Polyvinylidene difluoride
qPCR: Quantitative PCR
RT-PCR: Reverse transcription-polymerase chain reaction
SD: Standard deviation
SDS-PAGE: Sodium dodecyl sulfate-polyacrylamide gel electrophoresis
TBIL: Total bilirubin
TEM: Transmission electron microscopy
TNF-α: Tumour necrosis factor-α
Treg: Regulatory T cells
