# Supplementary figures and images for: Human liver stem cells attenuate concanavalin A-induced acute liver injury by modulating myeloid-derived suppressor cells and CD4+ T cells in mice
Source: Stem Cell Res Ther. 2019 Jan 11;10:22. doi: 10.1186/s13287-018-1128-2 (PMC6330470; doi:10.1186/s13287-018-1128-2)

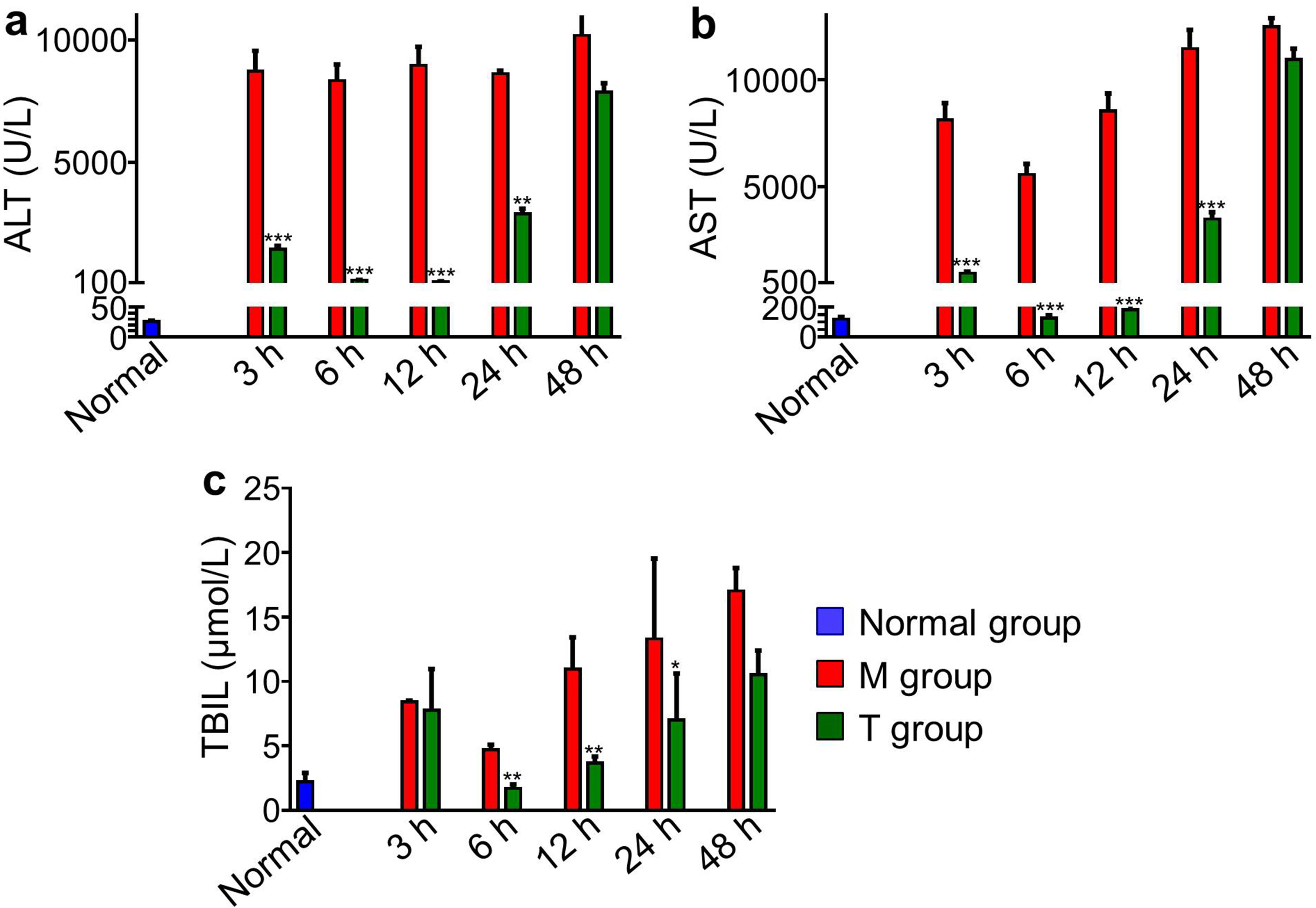

Supplement: Supplementary file 1 — Biochemical evaluation in the serum and the detection of tracing of transplanted cells. Supplementary data: supplementary materials and methods and supplementary figures. Supplementary Figure 1: Biochemical evaluation of (a) ALT, (b) AST, and (c) TBIL in the serum from groups of normal, M (ConA) and T (HYX1 + ConA) mice. Supplementary Figure 2: Tracing of transplanted cells. Supplementary Figure 3: Long-term protective effect of HYX1 on a 2nd ConA-induced liver injury. Supplementary Figure 4: Biochemical evaluation of ALT, AST and TBIL in the serum of mice with HYX1 transplantation alone. (ZIP 27721 kb) [file 13287_2018_1128_MOESM1_ESM.zip › Supplementary Figure 1.jpg]

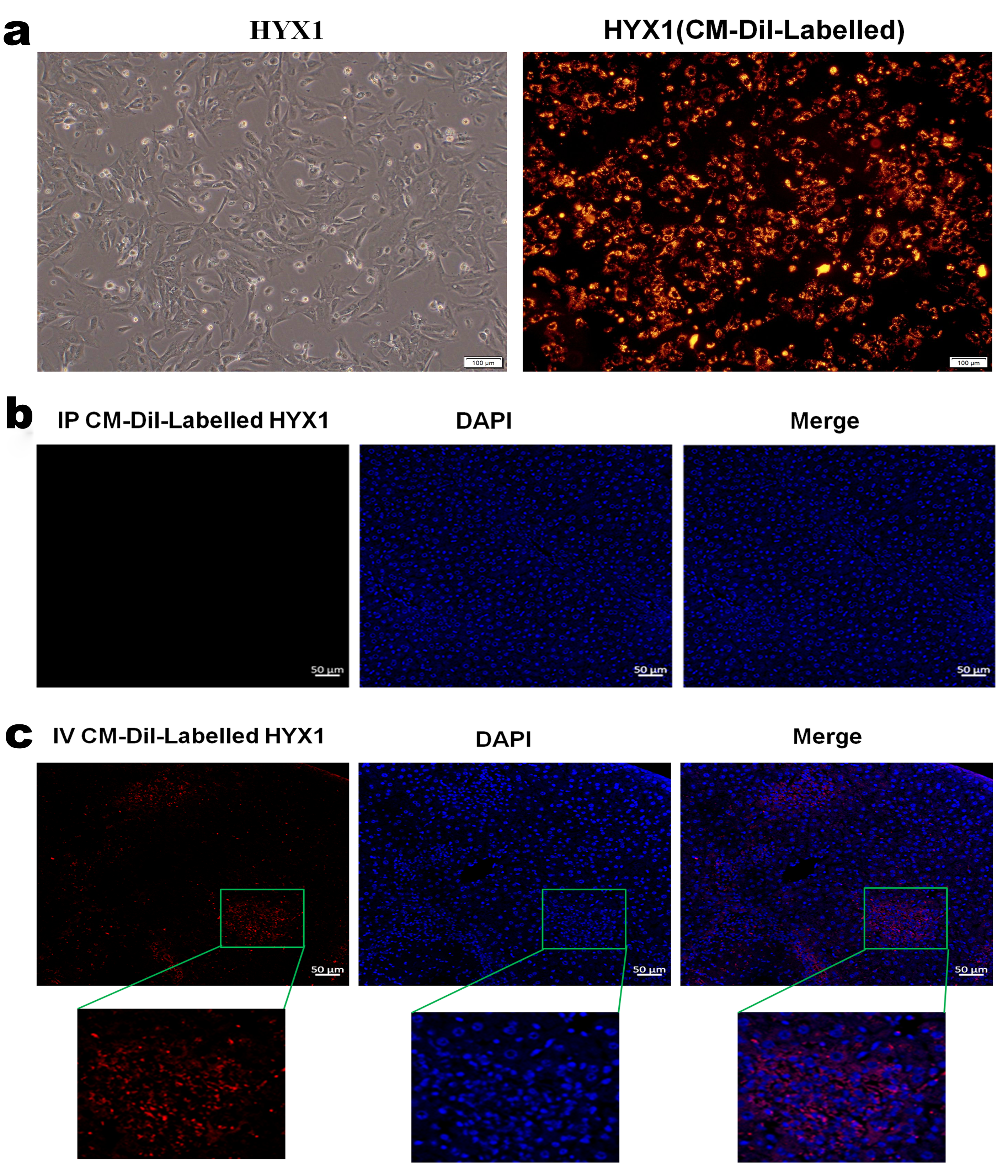

Supplement: Supplementary file 1 — Biochemical evaluation in the serum and the detection of tracing of transplanted cells. Supplementary data: supplementary materials and methods and supplementary figures. Supplementary Figure 1: Biochemical evaluation of (a) ALT, (b) AST, and (c) TBIL in the serum from groups of normal, M (ConA) and T (HYX1 + ConA) mice. Supplementary Figure 2: Tracing of transplanted cells. Supplementary Figure 3: Long-term protective effect of HYX1 on a 2nd ConA-induced liver injury. Supplementary Figure 4: Biochemical evaluation of ALT, AST and TBIL in the serum of mice with HYX1 transplantation alone. (ZIP 27721 kb) [file 13287_2018_1128_MOESM1_ESM.zip › Supplementary Figure 2.tif]

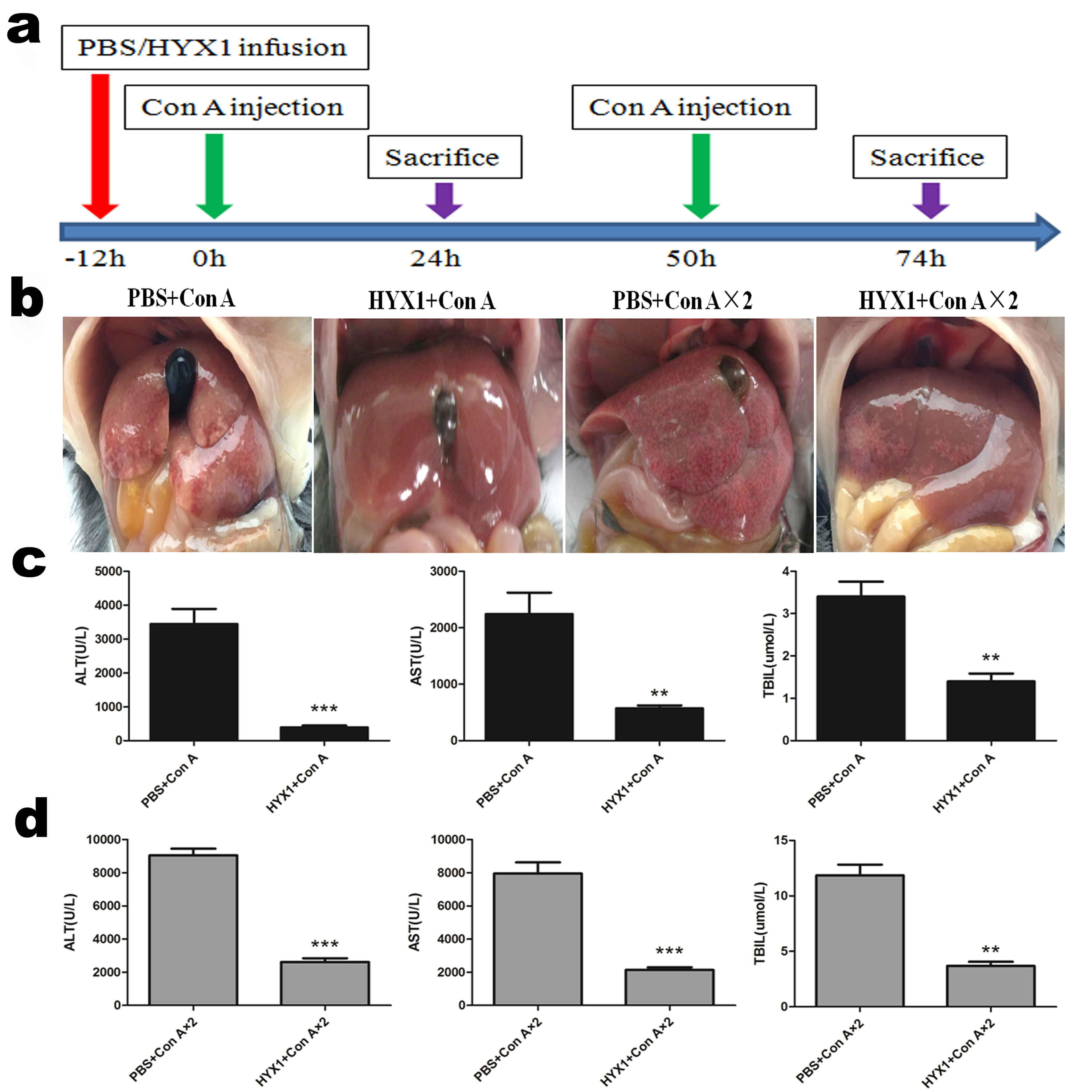

Supplement: Supplementary file 1 — Biochemical evaluation in the serum and the detection of tracing of transplanted cells. Supplementary data: supplementary materials and methods and supplementary figures. Supplementary Figure 1: Biochemical evaluation of (a) ALT, (b) AST, and (c) TBIL in the serum from groups of normal, M (ConA) and T (HYX1 + ConA) mice. Supplementary Figure 2: Tracing of transplanted cells. Supplementary Figure 3: Long-term protective effect of HYX1 on a 2nd ConA-induced liver injury. Supplementary Figure 4: Biochemical evaluation of ALT, AST and TBIL in the serum of mice with HYX1 transplantation alone. (ZIP 27721 kb) [file 13287_2018_1128_MOESM1_ESM.zip › Supplementary Figure 3.jpg]

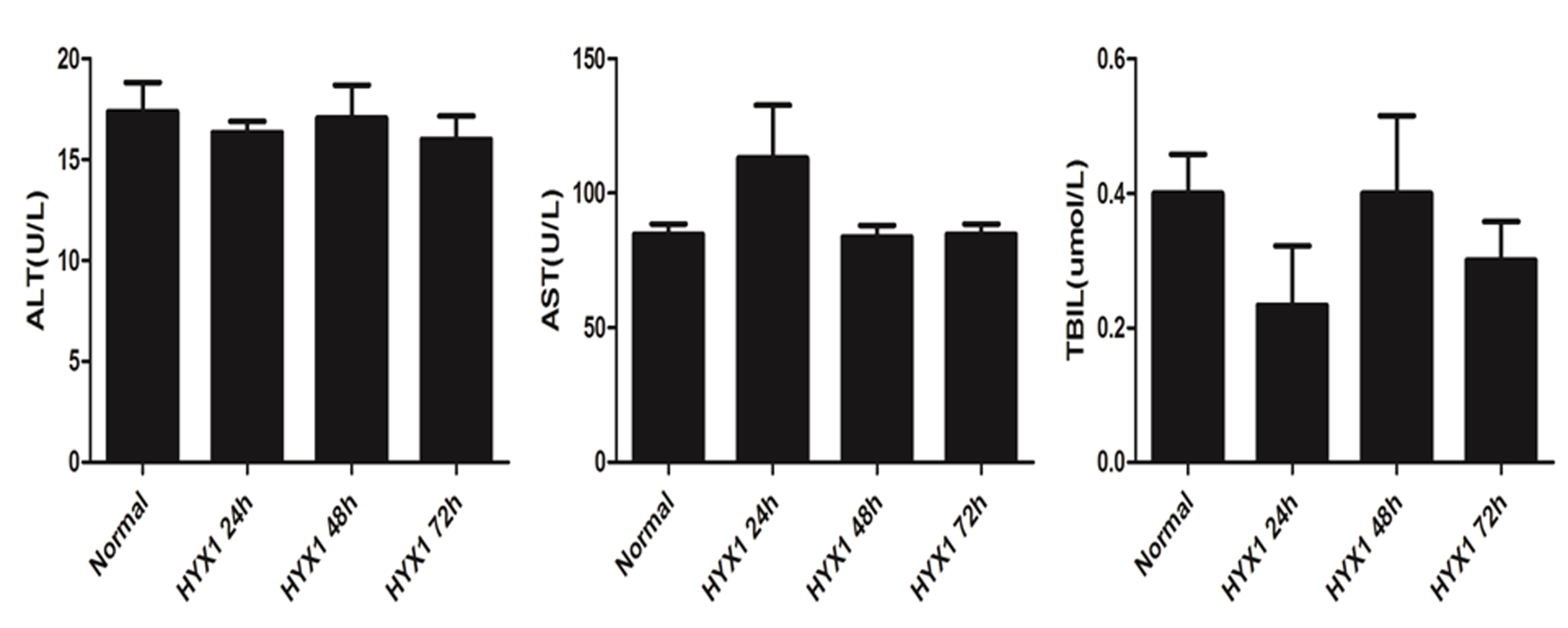

Supplement: Supplementary file 1 — Biochemical evaluation in the serum and the detection of tracing of transplanted cells. Supplementary data: supplementary materials and methods and supplementary figures. Supplementary Figure 1: Biochemical evaluation of (a) ALT, (b) AST, and (c) TBIL in the serum from groups of normal, M (ConA) and T (HYX1 + ConA) mice. Supplementary Figure 2: Tracing of transplanted cells. Supplementary Figure 3: Long-term protective effect of HYX1 on a 2nd ConA-induced liver injury. Supplementary Figure 4: Biochemical evaluation of ALT, AST and TBIL in the serum of mice with HYX1 transplantation alone. (ZIP 27721 kb) [file 13287_2018_1128_MOESM1_ESM.zip › Supplementary Figure 4.jpg]
