# Supplementary material for: Human liver stem cells attenuate concanavalin A-induced acute liver injury by modulating myeloid-derived suppressor cells and CD4+ T cells in mice
Source: Stem Cell Res Ther. 2019 Jan 11;10:22. doi: 10.1186/s13287-018-1128-2 (PMC6330470; doi:10.1186/s13287-018-1128-2)
Supplement: Supplementary file 1 — Biochemical evaluation in the serum and the detection of tracing of transplanted cells. Supplementary data: supplementary materials and methods and supplementary figures. Supplementary Figure 1: Biochemical evaluation of (a) ALT, (b) AST, and (c) TBIL in the serum from groups of normal, M (ConA) and T (HYX1 + ConA) mice. Supplementary Figure 2: Tracing of transplanted cells. Supplementary Figure 3: Long-term protective effect of HYX1 on a 2nd ConA-induced liver injury. Supplementary Figure 4: Biochemical evaluation of ALT, AST and TBIL in the serum of mice with HYX1 transplantation alone. (ZIP 27721 kb) [file 13287_2018_1128_MOESM1_ESM.zip › Supplementary data.doc]

**Human Liver Stem Cells Attenuates Concanavalin A-induced Acute Liver Injury via Modulating Myeloid-Derived Suppressor Cells and CD4^+^ T Cells Migration and Cytokines Secretion in Mice**

Yanzhen Bi^1⊥^, Jiannan Li^2⊥^, Yonghong Yang^3^, Quanyi Wang^3^, Quanquan Wang^4^, Xiaobei Zhang^3^, Guanjun Dong^5^, Yibo Wang^3^, Zhongping Duan^1^, Zhenfeng Shu^6^, Tongjun Liu^2^, Yu Chen^1,*^, Kai Zhang^2, *^, Feng Hong^3, *^

**Supplementary materials and methods**

**Cells**

**Culture of HYX1:**

The cells used were the 10 passage of human liver stem cells (HYX1). All cells were cultured in Dulbecco's modified Eagle's medium (DMEM) supplemented with 10% fetal bovine serum (FBS) and 1% penicillin/streptomicin (all from Gibco, Australia), and maintained in a humidified tissue culture incubator with 5% CO2 at 37°C. For all cells, upon reaching an 80% confluence, the cells were detached with 0.25% trypsin/EDTA (Gibco), centrifuged, and re-suspended in the same culture medium at the ratio of 1:2. Then they were suspended in PBS at 1 × 10^7^ cells/mL for transplantation.

The cells used in Supplementary Fig. 2 were labeled using CellTracker^TM^ CM-Dil Kit (C7000, Sigma-Aldrich) according to the manufacturer's recommendation to track the HYX1 engraftment in the liver tissues. The labeled cells were suspended in PBS at 1 × 10^7^ cells/mL for transplantation.

**Animal and Treatment**

In the present study, we established two more groups of intraperitoneal injection (n=10) and intravenous injection (n=10) to certify the engraftment of transplanted cells in the liver tissues in Supplementary Fig. 2. Mice were firstly injected with 12mg/kg ConA or equal volume of PBS via tail vein. Then they were treated with 1.0×10^6^ HYX1 (10 passage) via intraperitoneal or tail vein at 24 hours after ConA or PBS injection (n=5). The liver samples were obtained at 48 hours after transplantation.

To identify whether there are any long-term protective effect after HSLC/ConA due-administration, we established two more groups with PBS or HYX1 cells, respectively, 12 h before treatment with ConA (n=10) in Supplementary Fig. 3. Each group contained two different subgroups (n = 5) , PBS+Con A group, HYX1+ConA group, PBS+ConA×2 group and HYX1+ConA×2 group. Mice were sacrificed 24 h after the first ConA injection in PBS+ConA group and HYX1+ConA group, while they were sacrificed 24 h after the second ConA injection in PBS+ConA×2 group and HYX1+ConA×2 group (Supplementary Fig. 3a). The levels of ALT, AST and TBIL in the serum were detected by using an automated analyser (AU5800, Beckman Coulter, Brea, CA, USA).

In addition, 18 8-week-old male C57BL/6 mice were used for experiments to explore the changes caused by HYX1 transplantation alone in Supplementary Fig. 4. Mice were treated with 2.0×10^6^ HYX1 (10 passage) via intraperitoneal (n=15) and sacrificed 24 h, 48 h, 72 h after HYX1 transplantation (n=5). The remaining 3 mice did not do any treatment , as the normal control. The levels of ALT, AST and TBIL in the serum were detected by using an automated analyser (AU5800, Beckman Coulter, Brea, CA, USA).

**Supplementary figures**

**Figure S1** Biochemical evaluation of (a) ALT, (b) AST, and (c) TBIL in the serum from groups of normal, M (ConA) and T (HYX1 + ConA) mice. The serum ALT, AST and TBIL levels were markedly elevated in the ConA groups (M1, M2, M3, and M4 groups), and significantly decreased in the T1, T2, T3, and T4 (HYX1+ConA) groups, especially in the T2 and T3 groups compared with the ConA groups (*P* < 0.05). Data are presented as mean ± SEM (*n* = 6); compared with M group, **P* < 0.05, ***P* < 0.01, ***P* < 0.001, as determined by ANOVA tests or t-test; ns denotes p > 0.05.

**Figure S2** Tracing of transplanted cells. The cells were labeled with CellTracker^TM^ CM-DiI (red) prior to transplantation (a). Engraftment of labeled HYX1 in mouse liver, 2 days after transplantation. After 48 hours, transplanted cells (red) were detected in the liver of mice with liver injury in the intravenous group (c). While there were no transplanted cells detected in the liver of mice with or without liver injury in the intraperitoneal injection group (b).

**Figure S3** Long-term protective effect of HYX1 on a 2nd ConA-induced liver injury. (a) Schema of HYX1-pretreated acute liver injury transplants procedure. 2×10^6^ HYX1 were infused into mice via IP injection. All groups were sacrificed at indicated time points for sample collection. Zero hour represents the time point of the first ConA injection (12 mg/kg). Fifty hour represents the time point of the second ConA injection (12 mg/kg). (b) Representative images of the gross anatomy in mice challenged with ConA for 24 hours. (c, d) Serum ALT, ALT, and TBIL levels were measured as biomarkers of liver cell injury. Data are shown as the mean ± SEM (n = 5 replicates). ** p < 0.01, ** p < 0.001, as determined by independent-samples T test; ns denotes p > 0.05.

**Figure S4**  Biochemical evaluation of ALT, AST and TBIL in the serum of mice with HYX1 transplantation alone. There was almost no change in serum ALT, AST and TBIL in mice 24 h, 48 h, and 72 h after HYX1 transplantation alone, compared with normal mice. Data are presented as mean ± SEM; compared with normal group, **P* < 0.05, ***P* < 0.01, as determined by ANOVA tests; ns denotes p > 0.05.
